# NIR Irradiation Based on Low-Power LED Drive Module Design for Fat Reduction

**DOI:** 10.1155/2021/9992095

**Published:** 2021-08-12

**Authors:** Ki-Cheol Yoon, Kwang Gi Kim

**Affiliations:** ^1^Department of Biomedical Engineering, College of Medicine, Gachon University, 38-13, Dokjom-ro 3, Namdong-gu, Incheon 21565, Republic of Korea; ^2^Medical Devices R&D Center, Gachon University Gil Medical Center, 21, 774 beon-gil, Namdong-daero Namdong-gu, Incheon 21565, Republic of Korea; ^3^Department of Biomedical Engineering, College of Health Science, Gachon University, 191 Hambakmoero, Yeonsu-gu, Incheon 21936, Republic of Korea; ^4^Department of Health Sciences and Technology, Gachon Advanced Institute for Health Sciences and Technology (GAIHST), Gachon University, 38-13, 3 Dokjom-ro, Namdong-gu, Incheon 21565, Republic of Korea

## Abstract

In this study, we designed a low-power visible ray (V) drive module based on a light-emitting diode (LED) to initiate fat reduction using light source irradiation. A chemical phantom of muscle and fat was fabricated, and the performance of the proposed LED drive module was tested using this chemical phantom. The LED light source could reduce fat by irradiating the skin 4–5 cm deep. The device exhibits a negative feedback and parallel amplification to maintain a stable circuit based on low-power consumption. Muscles have a high-water content and low impedance, whereas fats have a low water content and significant salt content. Therefore, fat exhibits high impedance. Chemical phantoms were fabricated according to these impedance values, and the fat reduction effect using the LED circuits was analyzed. When the fat phantom was irradiated by the light source, the fat impedance lowered, and we confirmed that fat reduction could be obtained. This study is expected to be applicable to family medicine and weight management health care.

## 1. Introduction

The overweight and obese population is growing rapidly, and obesity is causing an increasing number of diseases [1]. Abdominal fat causes cerebrovascular disease [2]. The laser irradiation method of reducing abdominal fat can thermally decompose fat through the selective irradiation of adipose tissue [3]. The laser has a narrow bandwidth, and the beam is a straight path of light [4]. The laser generates heat through power consumption [4]. Therefore, intensive irradiation enables localized thermal decomposition of adipose tissue. However, the intense heat causes side effects, such as trauma, bleeding, burns, and pain [3]. In addition, lasers are expensive and have a high-power consumption.

Visible ray- (V) based light-emitting diode (LED) irradiation has demonstrated a lipolysis effect [5]. Light absorption by mitochondria promotes adenosine triphosphate (ATP) formation by consuming fat. Therefore, when the skin is irradiated with visible light with a wavelength of 400–900 nm, the light penetrates 4–5 cm deep into the skin and fat tissue is decomposed [5]. The LED has a wide beam width. Thus, wide and deep irradiation of adipose tissue is possible, widening the adipose tissue treatment area. It is also low cost in terms of unit cost and energy consumption and is resistant to heat [4].

Ultraviolet (UV) LED (400–700 nm) for direct irradiation has been reported to demonstrate a negative impact on the human body [6, 7]. However, V-LEDs (400–700 nm) are reported to have excellent bacteriological sterilization effects on the skin and are harmless to the human body [6–11].

In this study, we designed a low-power-consuming V-LED light source drive circuit for adipose tissue decomposition. To test the performance of the new light source drive module, experiments were performed using chemical phantoms of muscle and fat. In addition, animal experiments were conducted using mice to verify the phantom experiment results.

## 2. Analysis of Fat Reduction by LED Irradiation

**Figure 1:**
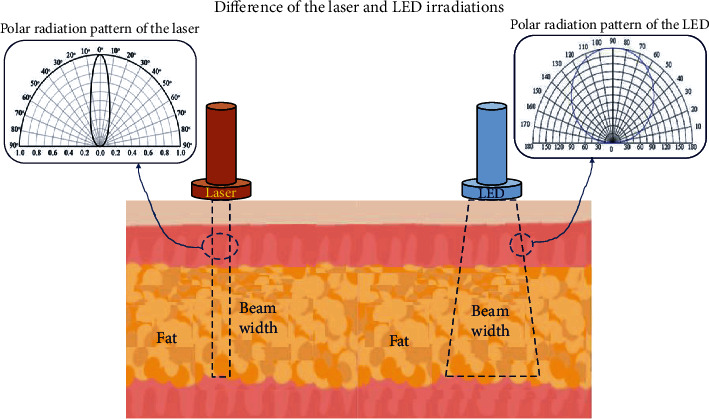
Difference between the laser and LED irradiation of tissues.

When a light source of 400–900 nm (V-LED) is applied to the skin, the beam is absorbed into the skin to a depth of 4–5 cm, as shown in Figure 1 [5].

**Figure 2:**
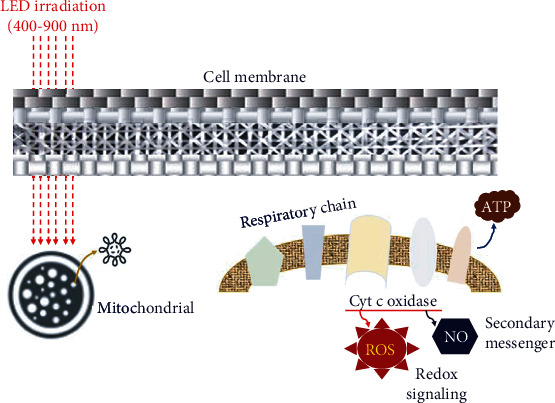
ATP generation of mitochondria following LED application.

The absorbed light penetrates the mitochondria to produce ATP very quickly, as shown in Figure 2. Specifically, the light source beam assists the production of ATP absorbed by cytochrome c oxidase and simultaneously releases active oxygen. Therefore, RNA and DNA are produced in cells [5].

**Figure 3:**
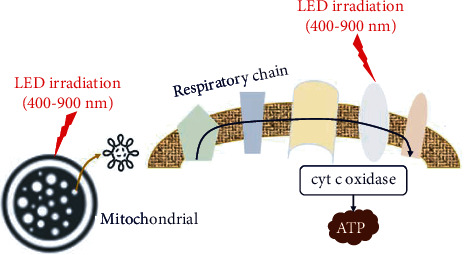
Emissions of oxidase and nitric acid from ATP.

In the Figure 3, the cytochrome c oxidase produces ATP with the help of oxygen. Nitric oxide (NO), produced in the mitochondria, interferes with ATP production by preventing cytochrome c oxidase from accepting oxygen [5]. When irradiated with a light source beam of 400–900 nm, cytochrome c oxidase releases nitric oxide and absorbs oxygen to help generate ATP in mitochondria [5]. Free radicals are also stimulated, increasing cells by RNA and DNA synthesis [5].

## 3. LED Drive Circuit Design

**Figure 4:**
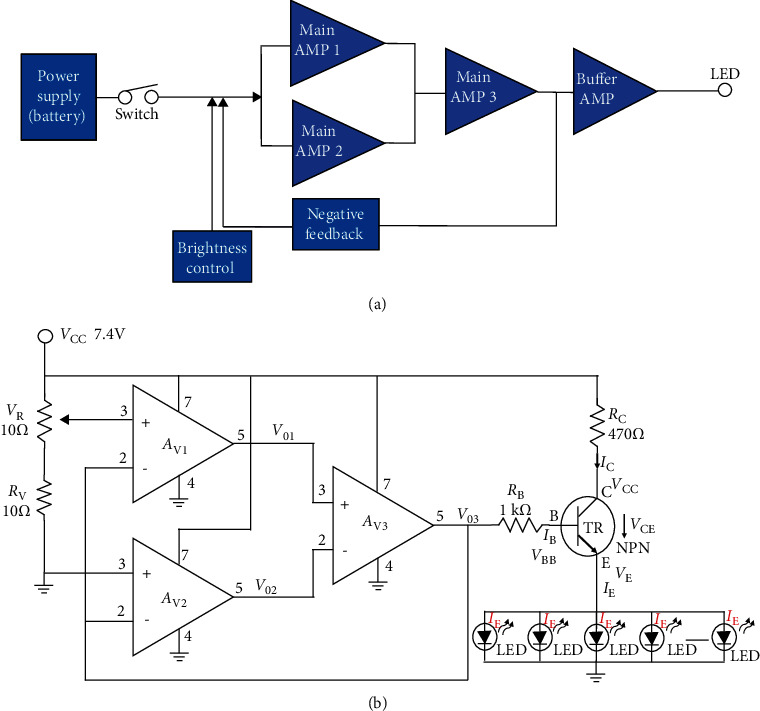
Proposed LED drive module: (a) structure and (b) circuit.

A parallel dual-stage amplifier was designed for low-power consumption and high stability in the LED drive circuit, and it generated a high current at low voltage, as shown in Figure 4. Therefore, it was possible to operate dual-structure V-LEDs efficiently.

Figure 4(a) shows the drive configuration diagram for the V-LED. It consists of a power supply, a switch, a light source brightness control, a main amplifier, a negative feedback control, and a buffer amplifier. Negative feedback was used to maintain a stable high current at low voltages and to minimize power consumption.

Figure 4(b) shows a circuit diagram for the V-LED. From the figure, *V*_R_ regulates the brightness of the light source, and *A*_V1_, *A*_V2_, and *A*_V3_ amplify the current through feedback. TR provides a buffer amplifier for the amplified current.

For circuit operation, it was assumed that the output voltage (*V*_o_) at the amplifier was equivalent to the input voltage (*V*_i_), as shown in

(1) $$\begin{array}{c} V_{\text{o}}=V_{\text{i}}. \end{array}$$

The negative amplification (*A*_V_), in which the input voltage at the amplifier is amplified, resulting in the output voltage (*V*_o_), was equal to 1 as

(2) $$\begin{array}{c} A_{\text{V}}=\frac{V_{\text{o}}}{V_{\text{i}}}=1. \end{array}$$

The current of the NPN transistor (TR) in the amplification was divided into the collector current (*I*_C_), base current (*I*_B_), and emitter current (*I*_E_), as shown in Equations (3)–(5). The current (*I*_B_) flowing from the collector (*I*_C_) and the current (*I*_B_) flowing from the base was the current (*I*_E_) flowing from the emitter. The current (*I*_E_) flowing to the emitter was the amplified current, and this current flowed to the LED, making the LED brightest.

(3) $$\begin{array}{c} I_{\text{C}}=\frac{V_{\text{CC}}-V_{\text{CE}}}{R_{\text{C}}}\;\left(\text{mA}\right), \end{array}$$

(4) $$\begin{array}{c} I_{\text{B}}=\frac{V_{\text{BB}}-V_{\text{CE}}}{R_{\text{B}}}\;\left(\text{mA}\right), \end{array}$$

(5) $$\begin{array}{c} I_{\text{E}}=I_{\text{B}}+I_{\text{C}}\;\left(\text{mA}\right). \end{array}$$

**Figure 5:**
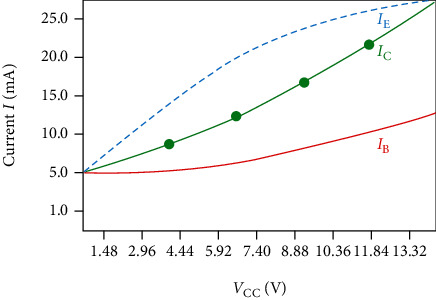
Operated current and voltage of LED drive module.

Figure 5 shows the simulation results for *I*_B_, *I*_C_, and *I*_E_ for the LED operation. As shown in the figure, *I*_B_, *I*_C_, and *I*_E_ are 6.7 mA, 14 mA, and 21 mA, respectively, when voltages *V*_CC_, *V*_BB_, and *V*_CE_ are 7.4 V, 0.7 V, and 6.7 V, respectively. At the same time, *V*_CC_ and *V*_BB_ represented the voltages flowing between the collector and base, and *V*_CE_ was the voltage flowing between the collector and base.

## 4. Experimental Results

### 4.1. Measurement Results

**Figure 6:**
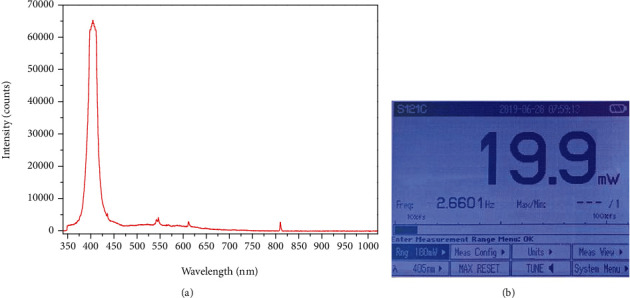
Measurement results for LED: (a) irradiation wavelength and (b) irradiation power.

**Figure 7:**
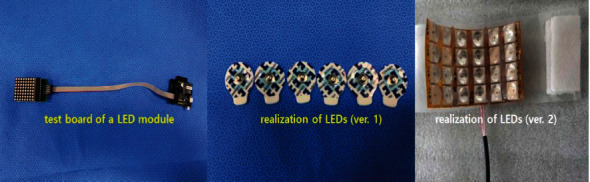
Fabrication of the proposed LED drive module.

To verify the operation of the proposed LED drive module, the irradiation wavelength and power are presented in Figure 6. From the figure, the irradiation wavelength and power are 406 nm and 19.9 mW, respectively.  Figure 7 shows the fabrication of the proposed LED drive module.

A chemical phantom was fabricated for performance testing of the proposed V-ray optical source drive module for fat reduction. To manufacture the phantom, the electrical inherent resistance values according to the tissue were required. The standard electrical conductivity (*ρ*) of muscle and fat is 0.05 s/m and 3.69 s/m, respectively [12]. The impedance (*Z*) and volume (*V*) corresponding to the muscle and fat can be calculated using Equations (6) and (7) [13].

(6) $$\begin{array}{c} Z=\rho \frac{L}{S}\;\left(\Omega \right), \end{array}$$

(7) $$\begin{array}{c} V=\rho \frac{L^{2}}{Z}\;\left(\text{mL}\right), \end{array}$$

where *L* and *S* are the length (mm) and dimension (mm), respectively, of the tissue and the units of *Z* and *V* are (*Ω*) and (mL), respectively. The impedance and volume of muscle tissue are 0.045 *Ω* and 27.8 mL, respectively, and the impedance and volume of fat tissue are 3.35 *Ω* and 27.5 mL, respectively. Chemical phantoms were fabricated with respect to these values.

**Figure 8:**
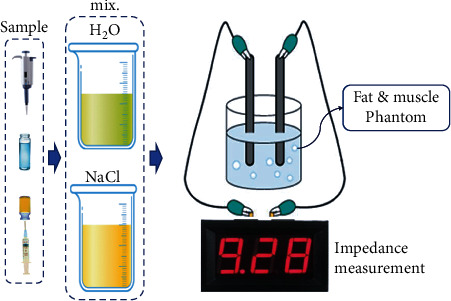
Process of the chemical phantom fabrication.

The manufacturing method combined 0.9% sodium chloride (NaCl) and oxidane (H_2_O) with N : 1, as shown in Figure 8. From the figure, the H_2_O and NaCl corresponding to the muscle were mixed at a 7 : 3 ratio. The impedance and volume were 0.045 *Ω* and 27.8 mL, respectively.

**Figure 9:**
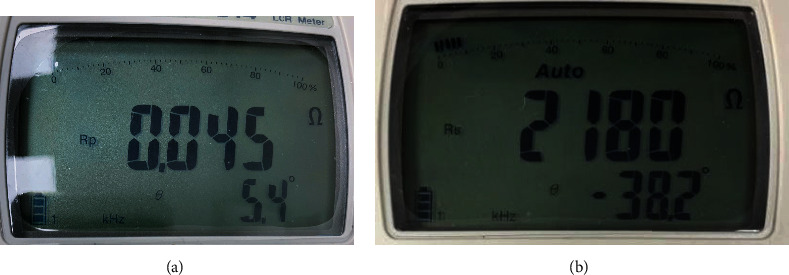
Measurement results for the characteristic impedance of fat and comparison between fat and water (instead of muscle) using chemical phantom (a) impedance of water (instead of muscle) and (b) impedance of fat.

The H_2_O and NaCl corresponding to the fat were mixed at an 8 : 2 ratio. The impedance and volume are 2180 *Ω* and 27.8 mL, respectively. The measurement results of the impedance values of muscle and fat for the mixing ratio are shown in Figure 9.

**Figure 10:**
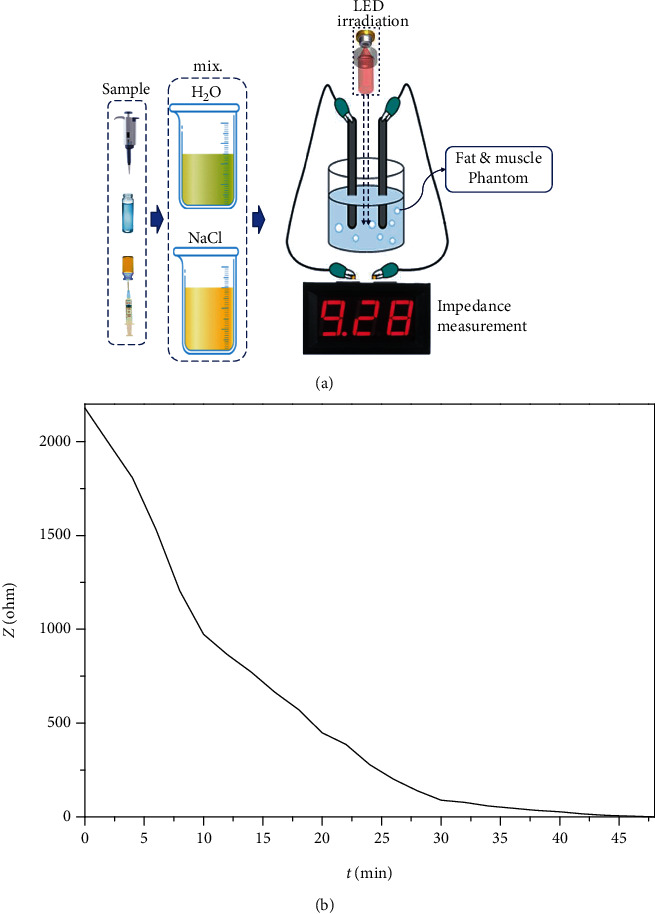
Measurement results for the characteristic impedance of fat reduction through the irradiation of LEDs (visible ray) using chemical phantom (a) irradiation of LEDs (visible ray) and (b) characteristic impedance (variation) of fat.

This is because muscles have a higher water content, and the fat contains more salt. The higher the moisture content, the lower the impedance, and the higher the salt content, the higher the impedance [12]. Irradiation of the fat chemical phantom resulted in lower impedance values and changed fat into moisture for use by muscles. LED irradiation sources were applied at 404 nm and 19.9 mW to the local chemical phantom, as shown in Figure 10. As shown in the figure, the irradiation time (*t*) was 48 min.

**Figure 11:**
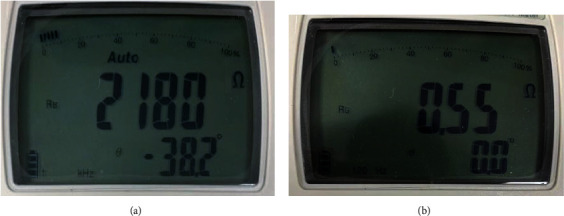
Measurement results for phantom fat (a) before and (b) after LED irradiation.

During the 48 min irradiation, the fat impedance was reduced to muscle impedance levels, as shown in Table 1. Fat impedance fell from 2180 *Ω* to 0.55 *Ω* after irradiation, and the measurement results are presented in Figure 11.

### 4.2. Animal Experiment Results

We used a C57BL/6 mouse in this study, which is the most commonly used variant in experiments related to obesity. The study was approved by the Institutional Animal Care and Use Committee of the Gachon University College of Medicine. Ninety adult mice were used in the experiment. The mice were divided into six groups, with each group comprising fifteen mice. The experimental conditions for the groups consisted of the following: (1) chow diet group: 6 groups of regular diets designated and fed by animal center, and (2) high-fat diet (HFD) group: 6 groups were fed food containing high fat.

**Figure 12:**
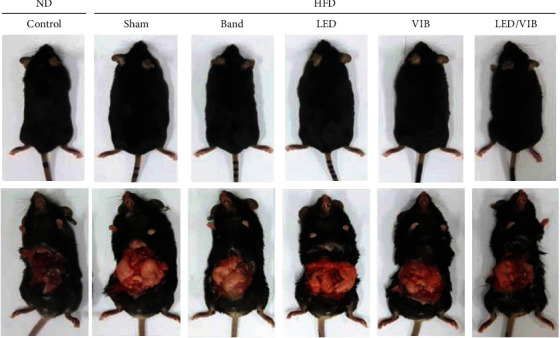
Gross appearance and abdominal fat comparison between experimental subjects.

Figure 12 compares the gross appearance and abdominal fat content of the subjects after eight weeks of the study period.

The gross appearance of the rats fed the HFD/Sham was large and fat in comparison with the rats fed a normal diet (ND). In addition, there was an increase in the gross abdominal fat of the HFD group compared to that in the ND group. However, the treatment group with LED/VIB exposure was shown to have a lesser amount of abdominal fat and was grossly leaner despite receiving an HFD.

**Figure 13:**
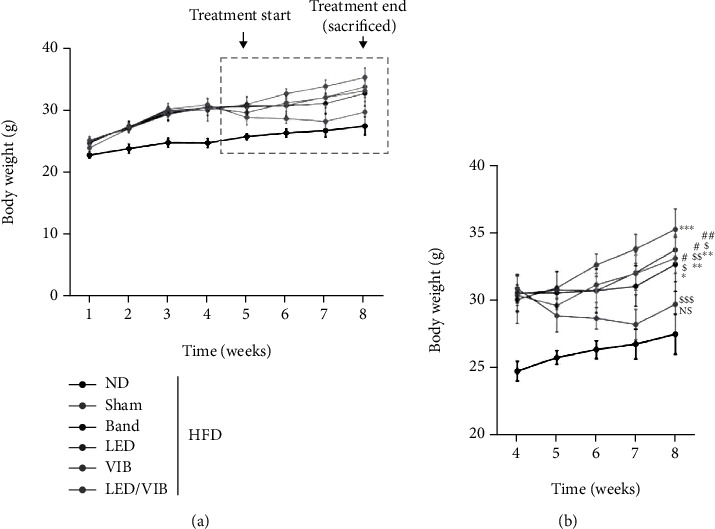
Changes in weight. (a) Overview of body weight changes. (b) Detailed view of the changes in body weight after four weeks of treatment.

The body weight of the ND group did not demonstrate significant variation after the fifth week as shown in Figure 13(a). The remaining rats received an HFD starting with their treatment from the fifth week, and the changes in body weight at the eighth week (after 4 weeks of treatment) were measured in each group.

A total of 48 LEDs (8 red LEDs and 40 near-infrared LEDs) were inserted into the device to allow only a special wavelength band to penetrate the fat layer, which is located in the third layer of the skin. The fabricated device was worn by the mouse for 1 h a day after four weeks of HFD. After one week of acclimatization and four weeks of feeding with HFD among the experimental groups, treatments were applied starting on the fifth week.

Body weight was the highest in the Sham diet group (*p* < 0.001 vs. ND). The LED/VIB treatment group showed the lowest weight, followed by the ND group, despite the ingestion of high-fat foods. This group also showed a statistically significant decrease in body weight (*p* < 0.001 vs. HFD) among the other treatment groups.

Body fat mass and lean mass were also analyzed after four weeks of HFD treatment.

**Figure 14:**
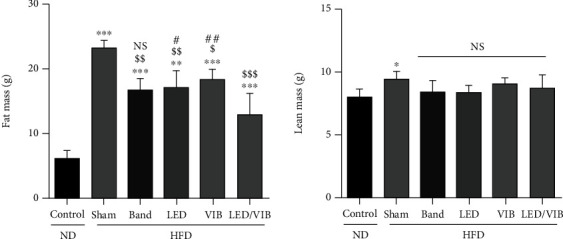
Body fat mass and lean mass comparison.

Body fat mass and lean mass content were also analyzed after the eight week study period. The body fat mass was shown to increase statistically significantly in those fed the HFD (Sham, red) when compared to the mice in the ND group (control, black), as shown in Figure 14. In the LED/VIB (LED/VIB, blue) treatment group, the body fat mass was significantly lower than that of the ND group despite the HFD intake. The lean mass, which is a measure of the nonfatty substances in the body, was significantly increased in the HFD group (Sham, red) compared to that in the ND group (control, black). However, there were no statistically significant differences among the other treatment groups.

**Figure 15:**
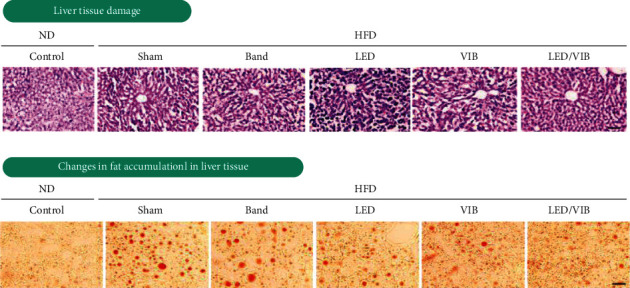
Histological analysis of mouse liver tissue after eight weeks of study.

Figure 15 shows the histological analysis of mouse liver tissue. Fat cells within the liver are stained with Oil Red O (ORO), where the degree of staining is proportional to the extent of cell differentiation. The size and amount of ORO-stained fat droplets increased significantly in the livers of the mice fed the HFD compared to those fed the ND, and there was no statistical difference in the livers of the mice receiving the band treatment. The LED/VIB treatment group showed a decrease in the size and amount of ORO-stained fat droplets despite the high-fat intake.

## 5. Conclusion

This article proposed an LED V-based high-efficiency generating circuit and module for reducing fat through irradiation. The designed LED circuit had low-power consumption, high efficiency, and high gain. The module combined stable operation with lower power consumption than that of laser diodes. The circuit was designed to be simple and small in size. LEDs have a wider beam width, lower cost, higher lifetime, and sufficient brightness compared to those of laser diodes.

Chemical fat phantoms were manufactured and measured to test the performance of the designed LED drive modules. Fat phantoms have a high impedance. However, irradiation by the LED light source caused electrolysis inside the phantom, reducing the impedance and changing it to moisture impedance. As the LED used in this experiment was a visible ray LED, which is harmless to the human body, it was determined to be suitable for direct irradiation of tissue. This study is expected to be applicable to family medicine and weight management health care.

## Figures and Tables

**Table 1:** Fat chemical phantom impedance values with time following visible ray irradiation.

*t* (min)	*Z* (*Ω*)	*t* (min)	*Z* (*Ω*)
0	2180	26	201.27
2	1992.31	28	139.51
4	1807.03	30	89.33
8	1529.11	32	77.75
10	1204.87	34	58.45
12	973.27	36	46.87
14	772.55	38	35.29
16	664.47	40	27.57
18	571.83	42	15.99
20	448.31	44	8.27
22	386.55	46	4.41

## Data Availability

No data were used to support this study.
